# Effects of Major Epigenetic Factors on Systemic Lupus Erythematosus

**DOI:** 10.29252/ibj.22.5.294

**Published:** 2018-09

**Authors:** Shirin Farivar, Fateme Shaabanpour Aghamaleki

**Affiliations:** Dept. of Molecular and Cell Biology, Faculty of Life Sciences and Biotechnology, Shahid Beheshti University G.C. Tehran, Iran

**Keywords:** DNA methylation, Epigenesis, Histones, microRNAs, Systemic lupus erythematosus

## Abstract

The pathogenesis of systemic lupus erythematosus (SLE) is influenced by both genetic factors and epigenetic modifications; the latter is a result of exposure to various environmental factors. Epigenetic modifications affect gene expression and alter cellular functions without modifying the genomic sequences. CpG-DNA methylation, histone modifications, and miRNAs are the main epigenetic factors of gene regulation. In SLE, global and gene-specific DNA methylation changes have been demonstrated to occur in CD4^+^ T-cells. Moreover, histone acetylation and deacetylation inhibitors reverse the expression of multiple genes involved in SLE, indicating histone modification in SLE. Autoreactive T-cells and B-cells have been shown to alter the patterns of epigenetic changes in SLE patients. Understanding the molecular mechanisms involved in the pathogenesis of SLE is critical for the introduction of effective, target-directed and tolerated therapies. In this review, we summarize the recent findings that highlight the importance of epigenetic modifications and their mechanisms in SLE.

## INTRODUCTION

Systemic lupus erythematosus (SLE) is a severe, chronic autoimmune disease that is characterized by the involvement of multiple organs including kidney, joints, and skin[1,2]. SLE is more prevalent in women (female:male ratio is 9:1); 70-90% of SLE patients are female. The increased frequency of SLE among women have been attributed to the effects of sex hormones on interferon (IFN)-α and toll-like receptor (TLR) as well as aberrant X chromosome inactivation or X chromosome dosage effects[3-7]. The main cause of this disease has not been determined yet, but it is thought to be multifactorial etiology, including the interaction of many genes, epigenetic factors viz DNA CpG methylation, histone tail modification, non-coding RNA (miRNAs, lncRNA, and siRNA), and environmental factors (sunlight, drugs, and infectious elements, especially Epstein-Barr virus)[4, 8-11].

The initial approaches, linkage analysis and candidate gene association studies, have identified 40 SLE-associated loci. The genome-wide association study could screen hundreds of thousands of single nucleotide polymorphisms (SNPs) and eight chromosome regions across genome in SLE patients[12-16]. The majority of SLE susceptibility genes encode the products involved in innate and adaptive immunity[17,18]. Among these varieties of elements for SLE etiology, today epigenetic factors are in the center of attention. Actually, epigenetics means beyond the genetics and includes some special changes in genome. It consist of three main modifications: DNA CpG methylation, histones modification (i.e. the addition of acetyl, methyl, and other chemical groups to some especial residue of histones), and lncRNA such as miRNA, in order to regulate mRNA expression[19]. Methylation modifications can occur through ultraviolet (UV) radiation, dietary contributions, and aging. Meanwhile, decrease in methylation level of several immune-related genes, e.g. TGAL (integrin alpha L chain, CD11a), CD40LG, TNFSF7 (CD70), KIR2DL4, and PRF1, can influence their expression in lupus T-cells. In addition, the increased H4 acetylation levels in monocytes, as one of the histone modifications, is frequently seen in SLE patients[20]. Several miRNAs, especially miR-21, miR-148a, and miR-126, can control the transcription of DNMT1 (DNA methyltransferase 1), a key component of DNA methylation[21].

A wide variety of studies have indicated epigenetic roles in SLE etiology. Epigenome-wide studies coupled with functional analysis of the epigenomic changes are able to determine novel important pathways and their mechanisms in the pathogenesis of some diseases like SLE. Given the importance of epigenetic factors, it can be expected that epigenetic therapy would be used and could be possible for SLE patients in the future, particularly when it is designed for target-specific regions within the genome. Therefore, in this review, we focus on the most important epigenetic factors and their mechanisms in SLE pathogenesis.

### DNA methylation in SLE patients

**Fig. 1:**
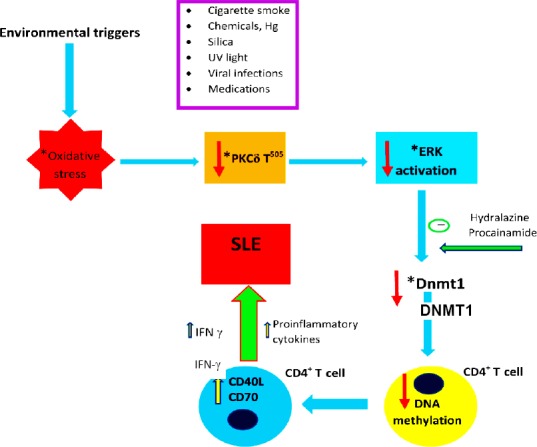
Schematic presentation of the proposed pathways in association with environmental factors that play a critical role in development of systemic lupus erythematosus (SLE). These processes take place inside the CD4^+^ T-cell. Hg, mercury; PKCδ T^505^, phosphorylated protein kinase C δ; ERK, extracellular signal-regulated kinase; DNMT1, DNA methyltransferase 1.

Methylation of DNA is one of the most important epigenetic modifications that can change gene expression by adding methyl group to the deoxycytosine base in CpG dinucleotide, to form deoxymethylcytosine. DNA methylation modifications can influence gene expression and play an important role in SLE (Fig. 1). MECP2 (methyl-CpG-binding protein 2), MBD2 (methyl-CpG-binding domain), and DNMT1 are the main parts of DNA methylation processes in different cells. Increased expressions of both MBD2 and DNMT1 in SLE patients could cause DNA hypermethylation and gene dysregulation[22,23]. According to different analyses of CpG methylation, including CD4^+^ T-cells, CD19^+^ B-cells, and CD14^+^ monocytes, done in various immune cell types of several SLE patients, it can be assumed that lupus patients exhibit more global DNA hypomethylation in CD4^+^ T-cell[24]. DNA demethylation and over-expression also occur in several genes as well as in TNFSF7 (CD70) that are normally methylated. TNFSF7 encodes CD70 on B-cell that contributes to antibody production. CD70 hypo-methylation and overexpression in T-cells of SLE patients cause IgG overexpression and production[25].

Environmental elements may affect epigenetic factors such as DNA methylation modifications. Exposure to UV light is associated with exacerbation of SLE. In addition, 73% of SLE patients have photosensitivity, which can be used as a diagnostic factor for this disease. Most cutaneous lesions occur in the light-exposed areas and can be triggered by sunlight exposure. UV light, especially UVB (290-320 nm), can reduce the expression of DNMT1 and that may cause T-cell auto-reactivity, accordingly inducing SLE[26,27]. Lymphocyte function-associated antigen-1 (LFA1) incorporates ITGAL and ITGB2 subunits, which is expressed on all leukocytes. Demethylation of the ITGAL gene promoter region requires an underlying mechanism for overexpression of LFA-1 on an auto-response set of T-cells in SLE patients. Indeed, LFA-1 overexpression, successively induces antibody production in B-cells, is believed to be concerned in T lymphocyte auto-reactivity in SLE[28,29].

DNA hypomethylation in CD4^+^ T-cells of SLE patients may happen under the influence of some chemical medications, together with 5-azacytidine, procainamide, and hydralazine, which could subsequently have impact on the expression of critical genes[30-32]. The 5-azacytidine is a cytosine analogue integrated into DNA during DNA replication and prevents DNA de novo methylation. Other DNA methylation inhibitors, like procainamide and hydralazine, are involved in CD4^+^ T-cells autoreactivity, in which autoreactive T-cell responds to MHC class II without the existence of exogenous antigen[33-35].

Patients with active lupus show lower methylated cytosine content (deoxymethylcytosine, about 4%) in their many genes of T-cells, as well as in ITGAL and TNFSF7. CD11a, perforin, and the KIR genes are also demethylated and overexpressed in patients with active, but not inactive, lupus. As a result, they can be used as a marker for distinguishing the disease activity[36].

DNA methylation can be one of the possible reasons for the SLE prevalence in females (∼90% of cases) through X chromosome inactivation. Female lupus patients display impaired DNA methylation on the inactive X chromosome. There are several X-linked genes that assist in SLE pathogenesis. DNA hypomethylation of CD40L on X chromosome plays an important role in the female predominance of SLE. Furthermore, the prevalence of SLE in women with Turner Syndrome (45, X0) is lower, but individuals with Klinefelter syndrome (47, XXY) have more potential for the progression of SLE[37-39]. It has been also shown that the hypermethylation of anti-inflammatory genes has a function in SLE pathogenesis[40]. HLA-DR alpha gene is hypermethylated in B-cells of SLE patients that may express a few amounts of HLA-DR antigen. The decreased expression of HLA-DR antigens and the HLA-DR alpha gene are associated with high anti-DNA antibody titers in patient’s serum[41].

### Effects of cell signaling on DNA methylation in SLE patients

DNA methylation can be regulated through several signaling pathways viz extracellular signal-regulated kinase (ERK) pathway (PKC [protein kinase c]→ Ras→ Raf→ MEK→ ERK). PKC is a member of protein kinase enzymes family and has catalytic domain in its c-terminal that involves in regulating a number of proteins by phosphorylating their serine and threonine residues in the ERK pathway with its c-terminal domain as a catalytic domain. PKC is located on 3p21.31 and contributes significantly to many cellular processes, including regulation of cell growth and programmed cell death, additionally in B-cell negative selection[42-44]. PKCδ is one of the members of PKC family. PKCδ phosphorylation is diminished in lupus patients. Strong evidence has shown that defective PKCδ phosphorylation is associated with ERK pathway deficiency and lower DNMT1 gene expression, thereby influencing DNA demethylation and up-regulation of the several genes including CD11A, CD70, CD40L, the pro-inflammatory cytokine IL-17A as well as several interferon-regulated genes[45]. PKCδ is also phosphorylated in response to other stimuli and activates ERK pathway. However, in patient with active lupus, PKCδis not properly phosphorylated in response to some chemical compounds, such as phorbol myristate acetate.. This phenomenon can be linked to the disturbance of ERK pathway and the low level of DNMT1 in CD4^+^ T-cells[45,46]. Another reason could be the increased level of reactive oxygen species and reactive nitrogen intermediates in lupus, which can induce the inappropriate phosphorylation of PKCδ through post-translational modifications[47,48]. A recent study has shown homozygous missense mutation in PKCδ (c. 1294G>T; p. Gly432Trp) of juvenile SLE patients. This mutation affects catalytic domain of PKCδ, which causes the loss of PKCδ function and the early onset of juvenile SLE. Missense mutations of PKCδ can also lead to Mendelian juvenile-onset SLE through increased B-cell proliferation with the resistance of B-cell to B cell receptor and Ca2^+^-dependent apoptosis[49].

Methylation modifications have recently been considered as a diagnostic and a prognostic marker for detecting response to therapy, and also the level of DNA methylation can be a potential biomarker for disease activity[50]. There are several major methylation changes in SLE patients that are shown in the Table 1.

**Table 1:** Some important genes and their methylation changes in SLE

Gene	Cell type	Methylation level	Effect in SLE	Ref.
*CD6*	CD3^+^ T-cells	+	Enhanced T-cell activation	[77,78]
*CREM*	CD3^+^ T-cells, CD4^+^ T-cells, Effector CD4^d+^ T-cells	+	Involved in the generation of DN T-cells and regulation of IL-2 and IL-17 in CD4^+^ T-cells	[77,78]
*FOXP3*	Treg	+	Reduced number and altered function of regulatory T-cells	[77]
*IL-2*	CD3^+^ T-cells, CD4^+^ T-cells, Effector CD4^+^ T-cells	++	Impaired production of regulatory T-cells, impaired function of cytotoxic CD8^+^ T-cells, effector CD4^+^ T-cell differentiation, and cytokine expression	[77,80]
*CDKN1A*	PBMCs	++	Potential effects on apoptosis and DNA repair	[77]
*SOCS1*	CD4^+xs^ T	++	Immune dysregulation	[20,77]
*TREX1*	PBMCs	++	Impaired exonuclease function and cytosolic DNA accumulation and the survival of autoreactive cells	[77,81]

+ Reduced DNA methylation;

++ increased DNA methylation; CD6, cluster of differentiation 6; FOXP3, forkhead-box-protein P3; CDKN1A, cyclin-dependent kinase inhibitor 1A; SOCS1, suppressor of cytokine signaling 1; PBMC, peripheral blood mononuclear cell; TREX1, three prime repair exonuclease 1

### miRNAs in SLE

miRNAs have an important role in SLE pathogenesis and progression through their functions in humoral and cellular immune system and immune cell development[21]. Recent studies have shown a different expression pattern of miRNAs in peripheral blood mononucleated cells (PBMCs) of SLE patients that indicate their contribution in SLE[51,52]. The miR-146a is located on the susceptible and predisposing locus 5q33.3 in SLE pathogenesis, which regulates IFN pathway and is underexpressed in the PBMCs of lupus patients. The miR-146a down-regulation can induce IFN pathway activity by targeting key proteins such as IRAK1 and STAT1. Moreover, an A/G SNP (rs5095329) within the promoter of miR-146a reduces promoter activity and its expression, which is correlated with SLE[53,54]. The miR-125a is another down-regulated miRNA in PBMCs of SLE patients that can indirectly controls RANTES expression through binding to KLF13 mRNA in activated T-cells[55]. miR-3148 is predicted to bind 3’-UTR region of TLR7 to decrease the expression of TLR7, as a main component of the innate immune system, that finally leads to a high inflammatory response in SLE patients. However, SNP G allele rs385839 in the 3’-UTR of the TLR7 gene can inhibit its binding to miR-3148 to increase the expression of TLR7 at mRNA and protein levels through reducing mRNA degradation. However, individuals who carry C allele of this SNP in the 3’-UTR of TLR7 show a decreased amount of TLR7 level, resulting in mRNA degradation[56]. On the other hand, miR-1246 expression decreases in the B-cell of SLE patients and is attached to the 3’-UTR of early B-cell factor 1 (EBF1) mRNA. Therefore, miR-1246 overexpression causes EBF1 mRNA degradation. EBF1 is an important player in activation, development, and division of B-cell by triggering the AKT signaling pathway[57]. miR-155 is another miRNA that is associated with SLE. It inhibits MYD88 and TAB2 to block inflammatory response. In contrast, it can increase the inflammatory response in macrophage and induces type 1 interferon signaling by targeting suppressor of cytokine signaling 1 (SOCS1)[58,59]. miR-let-7a (let-7a) stimulates immune system responses and an inflammatory component production, which contributes to SLE pathogenesis. Its overexpression may result in hyperplasia and a pro-inflammatory response. IL-6 contains a potential binding site for let-7a in its 3’-UTR and can lead to its production[60].

### Effects of miRNAs on DNA methylation in SLE patients

A previous study has shown that several miRNAs are regulated by epigenetic mechanisms, e.g. some miRNAs on the X chromosome can be influenced by DNA methylation during X chromosome inactiv-ation[61]. Some other studies have demonstrated that DNA demethylation occurs on the inactive X chromosome in female patients. Therefore, miRNAs such as miR-98, miR-188-3p, and let-7f-2, which are located on this chromosome, are highly expressed, which is likely due to the higher prevalence of SLE in females than males[62,63]. The overexpression of miR-148a and miR-126 (regulators of DNMT1) in CD4^+^ T-cells of SLE patints leads to global DNA hypomethylation. Each of these miRNAs directly inhibit DNMT1 through binding to its target 3’-UTR[61,64]. There are some miRNAs that affect DNMT1 indirectly. For instance, miR-29b reduces the expression of sp1, a DNMT1 transactivator, and miRNA-21 decreases the activity of Ras-MAPK-DNMT1 signaling pathway in T-cells of SLE patients[65]. Table 2 lists the miRNAs with their role in SLE (Table 2).

**Table 2:** miRNAs in the pathogenesis of SLE

miRNA	Target	Regulated process	Expression level in SLE	Ref.
miR-let-7a	IL-6	IL-6 induction	UP	[60]
miR-let-7c	Blimp1, SOCS1	Activation of DCs	UP	[82]
miR-125a	KLF13	CCL5 induction in T-cells	DOWN	[55]
miR-146a	TRAF6, IRAK1, TRAF6, IRAK1, IRAK2, IRF5, STAT1	NFκB mediated inflammatory response RIG-I-dependent anti-viral pathway Type I IFN induction and signaling	DOWN	[53,54,83]
miR-150	SOCS1	Renal fibrosis	DOWN	[84]
miR-155	MyD88, TAB2 SOCS1, PP2Ac	TLR/IL-1 inflammatory pathway, Type I IFN signaling, IL-2 induction	UP	[59,85-87]
miR-17~92	PTEN, BimRora, PHLPP2	The proliferation of lymphocytes, Differentiation, and function of Tfh cells	UP	[65,88]
miR-23b	TAB2, TAB3, IKKα	IL-17, TNF-α, IL-1β signaling	DOWN	[89]
miR-30a	Lyn	Activation of B-cells	UP	[90]
miR-31	RhoA	IL-2 induction in T-cells	DOWN	[91]

UP, up-regulated; DOWN, down-regulated

### Histones modifications in SLE patients

Acetylation, phosphorylation, and methylation of histones tails are the most important changes among histones modifications. A variety of enzymes and complexes of proteins, including lysine acetyl-transferases, histone deacelytase, lysine methyltransferases, and lysine demethylases, are responsible for creating specific epigenetic codes (histone methylation, histone acetylation, etc.). Epigenetic codes are extremely conserved and determine the phenotype and function of cells and tissues. Any destruction of histone codes contributes to the etiology of many diseases, especially autoimmune disorders like SLE. One of the histone code modifications occurs on lys9 and lys27 of H3 that can trigger chromatin compacting and gene silencing.

SLE patients have indicated decreased acetylation of whole histones and H3K9 methylation in their CD4^+^ T-cells. Promoter region of hematopoietic progenitor kinase 1 (HPK1) in CD4^+^ T-cells has trimethylated lys27 H3 that leads to the inhibition of HPK1 expression and assist in auto-immune response in SLE[66]. Down-regulation of HPK1 also results in the enhancement of T-cell level and the production of INF-γ and [54,67].

Evidence has shown that di-methylation of lys4 H3 increases the promoter of TNFSF7 (CD70) gene in SLE CD4^+^ cells, which is correlated with disease activity[68]. H3K4me3 level has also been demonstrated to elevate in some candidate genes, including PTPN22 and LRP1B, in PBMC of SLE patients[69].

Histone changes usually have relationship with DNA methylation; a methylated region of DNA has deacetylated histone that helps gene silencing and chromatin compacting. As an example, down- regulation of IL-2 expression with mediating cAMP-responsive element modulator in T-cells of lupus patients happens through both histone deacetylation and DNA hypermethylation[70]. Unusual histone acetylations have been observed near to IL-17 gene locus in T-cell of lupus patients[71].

Neutrophil extracellular traps (NETs) are chromatin structures that release from apoptotic blebs during apoptosis in many diseases like SLE[72]. Histones, which are secreted in NETosis process, are two to three times more acetylated on H2B-K12 and H4-K8, K12, and K16 and methylate on H3-K27 in SLE patients in contrast to healthy individuals, determining the association of the histones acetylation and methylation with apoptosis, NETs, and SLE[73,74]. Another histone modification in lupus patients is H3 and H4 acetylation, which is correlated with TNF-α locus and causes TNF-α hyper-expression in monocytes of SLE patients[75].

A genome-wide analysis and a global H4 acetylation analysis by ChIP-chip methodology show that the level of H4ac enhances in monocytes of SLE patients[71]. It has been suggested that 63% of genes having an abundance level of H4ac are associated with interferon regulatory factor 1 and the SLE pathogenesis[76]. Several major histone modifications in SLE are presented in Table 3.

**Table 3:** Most important histone modifications within some genes in SLE

Gene	Modification	Cell type	Effect in SLE	Ref.
*CD8A, CD8B*	H3K18 deacetylation, H3K27 trimethylation in DN T-cells	CD4^+^ T-cells, CD8^+^ T-cells, DN T-cells	Generation of CD3^+^ CD4^−^ CD8^−^ DN T-cells	[79]
*ITGAL*	Reduced H3K27 tri-methylation through histone demethylase	CD4^+^ T-cells	Increased T-cell-mediated inflammation	[92]
*TNF*	H3 acetylation	Monocytes	Increased monocyte maturation and pro-inflammatory cytokine expression	[75]
*IL-2*	H3K18 deacetylation, H3K27 trimethylation	CD3^+^ T-cells, CD4^+^ T-cells, effector CD4^+^ T-cells	Impaired production of regulatory T-cells, reduced activation-induced cell death, and longer survival of autoreactive T-cells; impaired function of cytotoxic CD8^+^ T-cells, effector CD4^+^ T-cell differentiation, and cytokine expression	[93,94]

Immune cells from SLE patients are characterized by a dysregulated gene expression profile. A significant proportion is caused by epigenetic alterations. A vast number of studies have identified several epigenetics factors, including miRNA, DNA methylation, and histone modification that are involved in SLE pathogenesis. This review tried to summarize some important areas of molecular pathogenesis of SLE. The presented data may facilitate the identification of new markers with possible application in diagnosis, prognosis, monitoring, or treatment of the disease.
